# Transport-Mediated Oxaliplatin Resistance Associated with Endogenous Overexpression of MRP2 in Caco-2 and PANC-1 Cells

**DOI:** 10.3390/cancers11091330

**Published:** 2019-09-08

**Authors:** Riya Biswas, Piyush Bugde, Ji He, Fabrice Merien, Jun Lu, Dong-Xu Liu, Khine Myint, Johnson Liu, Mark McKeage, Yan Li

**Affiliations:** 1 School of Science, Auckland University of Technology, Auckland 1010, New Zealand (R.B.) (P.B.) (J.H.) (F.M.) (J.L.); 2 AUT-Roche Diagnostics Laboratory, School of Science, Auckland University of Technology, Auckland 1010, New Zealand; 3 School of Interprofessional Health Studies, Auckland University of Technology, Auckland 0627, New Zealand; 4 College of Life Sciences and Oceanography, Shenzhen University, Shenzhen 518071, China; 5 College of Food Engineering and Nutrition Sciences, Shaanxi Normal University, Xi’an 710119, China; 6 The Centre for Biomedical and Chemical Sciences, School of Science, Faculty of Health and Environmental Sciences, Auckland University of Technology, Auckland 1010, New Zealand; 7 Department of Pharmacology and Clinical Pharmacology, University of Auckland, Auckland 1023, New Zealand (K.M.) (M.M.); 8 Auckland Cancer Society Research Centre, University of Auckland, Auckland 1023, New Zealand; 9 Department of Pharmacology, School of Medical Sciences, University of New South Wales, Sydney, NSW 2052, Australia

**Keywords:** oxaliplatin, gastrointestinal cancer, multidrug resistance protein 2 (MRP2)

## Abstract

Our recent publications showed that multidrug resistance protein 2 (MRP2, encoded by the ABCC2 gene) conferred oxaliplatin resistance in human liver cancer HepG2 cells. However, the contribution of MRP2 to oxaliplatin resistance remains unclear in colorectal and pancreatic cancer lines. We investigated the effects of silencing MRP2 by siRNA on oxaliplatin accumulation and sensitivity in human colorectal cancer Caco-2 cells and pancreatic cancer PANC-1 cells. We characterized the effects of oxaliplatin on MRP2 ATPase activities using membrane vesicles. Over-expression of MRP2 (endogenously in Caco-2 and PANC-1 cells) was associated with decreased oxaliplatin accumulation and cytotoxicity, but those deficits were reversed by inhibition of MRP2 with myricetin or siRNA knockdown. Silencing MRP2 by siRNA increased oxaliplatin-induced apoptotic rate in Caco-2 and PANC-1 cells. Oxaliplatin stimulated MRP2 ATPase activity with a concentration needed to reach 50% of the maximal stimulation (EC_50_) value of 8.3 ± 0.7 µM and Hill slope 2.7. In conclusion, oxaliplatin is a substrate of MRP2 with possibly two binding sites, and silencing MRP2 increased oxaliplatin accumulation and cytotoxicity in two widely available gastrointestinal tumour lines (PANC-1 and Caco-2).

## 1. Introduction

Oxaliplatin-based chemotherapy has been widely adopted in the clinic as a standard and preferred chemotherapy regimen for treating gastrointestinal cancer, including colorectal and pancreatic cancer [1,2,3]. Oxaliplatin has been used in combination with 5-fluorouracil and folinic acid (FOLFOX) or capecitabine (XELOX) as a first-line treatment for colorectal cancer [1]. Oxaliplatin has also been widely used in combination with 5-fluorouracil, irinotecan, and folinic acid (FOLFIRINOX) or gemcitabine and capecitabine (GEMOXEL) in treating advanced pancreatic cancer [1,2,4]. However, tumour resistance to oxaliplatin-based chemotherapy is a major clinical limitation. Several mechanistic studies demonstrated that cellular accumulation of oxaliplatin is a determinant of cellular sensitivity in vitro [5,6,7], suggesting platinum accumulation may be an important factor for determining clinical efficacy and tumour resistance. Accumulating evidence suggests that both ATP-binding cassette transporters (ABC transporters) and solute carrier transporters (SLC transporters) transport oxaliplatin into and out of cells [8,9,10]. Some of these membrane transporters may play important roles in oxaliplatin disposition and accumulation and thus platinum-based cytotoxcity [8,11,12,13,14]. 

We previously found that MRP2 (an ABC transporter encoded by the ABCC2 gene) transports oxaliplatin using energy derived from ATP hydrolysis in a membrane vesicle study [15]. It has been reported that manipulating the expression or function of MRP2 could attenuate oxaliplatin resistance in gastrointestinal cancer cells [16,17,18,19]. We recently demonstrated that over-expression of MRP2 endogenously in HepG2 cells was associated with decreased oxaliplatin accumulation and cytotoxicity, but those deficits were reversed by inhibition of MRP2 with a model MRP2 inhibitor myricetin (60 μM) or siRNA knockdown [20]. Our study of mice bearing tumour xenografts of HepG2 demonstrated that inhibiting MRP2 with myricetin caused increased in vivo sensitisation to oxaliplatin antitumour activity with little or no increase in toxicity [20]. In our analysis of a clinical gene expression dataset, we showed that ABCC2 was the only one of 18 oxaliplatin transporter candidate genes with differential tumour expression between CRC patients who did or did not respond to oxaliplatin-based chemotherapy [20]. However, the direct evidence of the contribution of MRP2 to determining oxaliplatin accumulation and sensitivity in models of human CRC and pancreatic cancer remains unclear, despite oxaliplatin-based chemotherapy being of major clinical importance for treatment of both malignancies. 

In this study, we aimed to identify human colorectal and pancreatic cancer lines with transport-mediated resistance to oxaliplatin associated with endogenous overexpression of MRP2. We determined if silencing MRP2 by siRNA could increase oxaliplatin accumulation and cytotoxicity in these human CRC and pancreatic cancer cell lines (i.e., Caco-2 and PANC-1). As MRP2 has been recently reported to protect GI cancer cells from apoptosis [16], there is also the possibility that dual-role mechanisms might be identified. To better understand the mode of oxaliplatin transport mediated by MRP2, we investigated the effect of oxaliplatin on MRP2 ATPase activities. 

## 2. Results

### 2.1. Caco-2 and PANC-1 Cells Endogenously Overexpress MRP2

**Figure 1:**
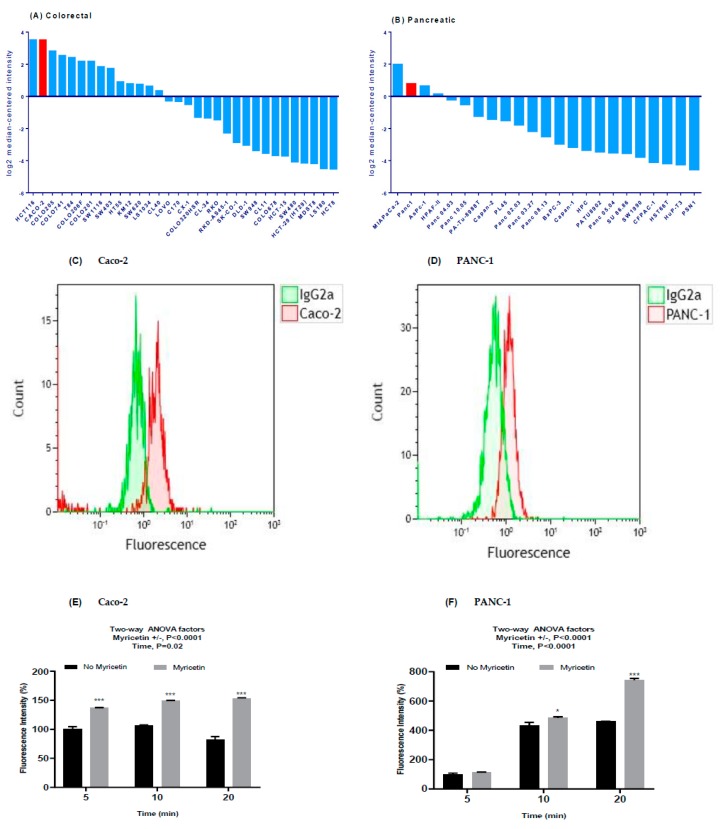
Functional overexpression of multidrug resistance protein 2 (MRP2) in human colorectal cancer Caco-2 cells and pancreatic cancer PANC-1 cells. (**A**,**B**) show high ABCC2 mRNA expression in Caco-2 and PANC-1 cells (both in red color) compared to other colorectal and pancreatic cancer cell lines from Wagner dataset stored in ONCOMINE (https://www.oncomine.org). (**C**,**D**) show MRP2 protein detected in representative flow cytometry histogram of cell surface staining using the anti-MRP2 primary antibody (red) and isotype control IgG2a (green) on Caco-2 and PANC-1 cells. Both the primary antibody and isotype control were labelled with Alexa Fluor 488 secondary antibody. The x-axis is the fluorescence signal intensity displayed in a liner log scale. Functional expression of MRP2 detected by CDCF accumulation in Caco-2 cells (**E**) and PANC-1 cells (**F**) at different time points in the presence and absence of 60 μM myricetin. All data are normalized to the fluorescence intensity determined at 5 min in the absence of myricetin. The bar represents the mean and standard errors of the mean values from at least three independent experiments. Asterisks are *p* values (*, *p* < 0.05; ***, *p* < 0.001) for differences at each time point from Sidak post-tests that followed a two-way analysis of variance (ANOVA).

Figure 1A,B show high *ABCC2* mRNA expression in Caco-2 (ranked 2/32) and PANC-1 (ranked 2/23) cells compared to other colorectal and pancreatic cancer cell lines based on Wagner dataset stored in ONCOMINE (https://www.oncomine.org). To confirm the surface protein expression of MRP2 on Caco-2 and PANC-1 cells, the cells were assessed by staining with anti-MRP2 primary and control isotype IgG2a antibody. Figure 1C,D show increased fluorescence with the MRP2 antibody stained Caco-2 and PANC-1 cells (three- and two-fold) compared to isotype control antibody treated samples, suggesting the expression of MRP2 protein on the surface of Caco-2 and PANC-1 cells. Furthermore, we investigate the MRP2 efflux activity in Caco-2 and PANC-1 cells by determining the cellular accumulation of a fluorescent MRP2 substrate, 5(6)-carboxy-2,’7’-dichlorofluorescein (CDCF) in the presence and absence of an MRP2 inhibitor myricetin at different time points. Myricetin significantly (*p* < 0.01) increased the steady-state accumulation of CDCF in both Caco-2 cells (Figure 1E) and PANC-1 cells (Figure 1F). Taken together, these results suggested that Caco-2 and PANC-1 cells endogenously overexpress MRP2.

### 2.2. Reduced MRP2 Expression in ABCC2-siRNA-Transfected Cells

**Figure 2:**
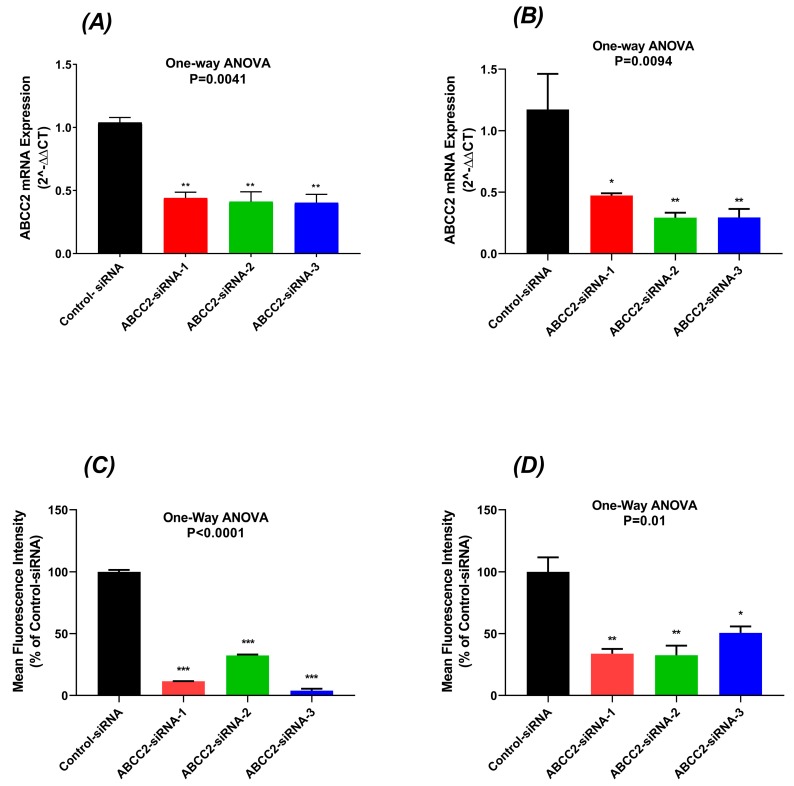
ABCC2 expression level at mRNA level in Caco-2 (**A**) and PANC-1 cells (**B**) transfected with control and ABCC2-siRNAs. Relative ABCC2 mRNA expression was detected by quantitative real-time PCR. ABCC2 mRNA expression was normalised to the reference gene GAPDH and relative quantitation of gene expression was calculated using the comparative threshold cycle method (2^−ΔΔCT^). All data were expressed as mean and standard errors of the mean from three independent experiments. The cell surface protein expression of MRP2 is presented as a mean percentage of control in (**C**) Caco-2 and (**D**) PANC-1 cells. The bar represents the mean and standard errors of the mean values from at least three independent experiments. Asterisks are *p* values (*, *p* < 0.05; **, *p* < 0.01; ***, *p* < 0.001) from Dunnett’s post hoc test that followed one-way ANOVA for comparisons of all ABCC2-siRNA samples to the negative control.

Caco-2 and PANC-1 cells were used for ABCC2 gene expression knockdown studies. The expression of mRNA transcripts of the MRP2 gene (ABCC2) was lower in ABCC2-siRNA subtypes transfected cells compared with the control-siRNA transfected cells. In Caco-2 cells, the mRNA transcripts of the MRP2 gene were significantly decreased by 56%, 59%, and 60% in ABCC2-siRNA-1, ABCC2-siRNA-2, and ABCC2-siRNA-3 transfected Caco-2 cells (*p* < 0.01), respectively (Figure 2A). In PANC-1 cells, ABCC2 mRNA expression was decreased by 50%, 70%, and 72% in ABCC2-siRNA-1, ABCC2-siRNA-2, and ABCC2-siRNA-3 transfected PANC-1 cells (*p* = 0.009), respectively (Figure 2B).

72 h after siRNA transfection, the surface expression of the MRP2 transporter in cells was measured by cell surface staining. The MRP2 surface expression was decreased by 70–89% in Caco-2 cells (*p* < 0.0001) (Figure 2C) and 50–60% in PANC-1 cells (*p* = 0.01) (Figure 2D). These results showed that transfection of stealth ABCC2-siRNAs results in significant decrease of the MRP2 mRNA and surface protein expression in both Caco-2 and PANC-1 cells.

### 2.3. ABCC2-siRNA Sensitized GI Cells to Oxaliplatin Growth Inhibition

**Figure 3:**
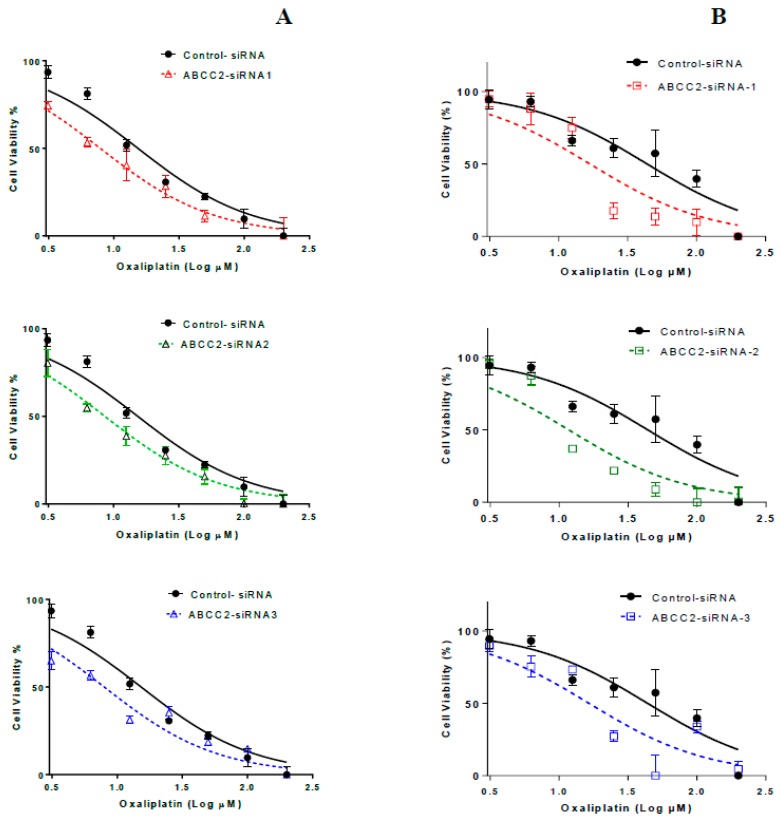
Representative oxaliplatin-induced inhibition of growth of Caco-2 (**A**) and PANC-1 (**B**) cells transfected with control and ABCC2-siRNA. Symbols are means and standard errors of the mean [*n* = 3]. Solid and dashed lines are non-linear regression fits (Y = Bottom + (Top − Bottom)/(1 + 10^(LogIC50 − X)) to the data.

After transfection with ABCC2-siRNA subtypes for 48 h, Caco-2 cells were treated with various concentrations of oxaliplatin (3−200 µM) for 2 h and the 72-h cytotoxicity of oxaliplatin was determined using an MTT [3-(4,5-dimethylthiazol-2-yl)-2,5-diphenyltetrazolium bromide] assay. Oxaliplatin inhibited the growth of negative control and ABCC2-siRNA transfected Caco-2 and PANC-1 cells in a dose-dependent manner. As shown in Table 1, the IC50 values were decreased by almost 50% in ABCC2-siRNA transfected Caco-2 cells and by 50−66% in ABCC2-siRNA transfected PANC-1 cells (Figure 3 and Table 1). These results indicated that transfection of siRNAs increased the sensitivity of cells to oxaliplatin. 

### 2.4. ABCC2-siRNA Increased CDCF and Platinum Accumulation in GI Cancer Cells

The functional activity of the MRP2 transporter was confirmed by steady-state CDCF accumulation in ABCC2-siRNA-transfected Caco-2 and PANC-1 cells. Compared with control-transfected cells the mean fluorescence in ABCC2-siRNA-transfected Caco-2 and PANC-1 cells were increased (Table 2). The increased fluorescence intensity in transfected cells suggested an increased accumulation of MRP2 model substrate CDCF in cells and decreased MRP2-mediated efflux activity.

Cellular accumulation of oxaliplatin-derived platinum was determined after silencing the ABCC2 in transfected cells. The platinum accumulation was significantly increased in ABCC2-siRNA-transfected cells as compared to negative control transfected cells. After 2 h exposure to 25 µM and 100 µM of oxaliplatin, the platinum accumulation in negative control-siRNA Caco-2 cells was 5.8 ± 0.3 and 105.2 ± 0.4 pmol/mg of protein respectively. Silencing MRP2 by ABCC2-siRNA-2 and ABCC2-siRNA-3 resulted in a two-fold increase in the platinum accumulation after 2 h exposure to 25 µM of oxaliplatin in ABCC2-siRNA Caco-2 cells (Table 2). The cellular platinum accumulation after 2 h exposure to 100 µM of oxaliplatin significantly increased in ABCC2-siRNA-2- or ABCC2-siRNA-3-transfected Caco-2 cells (Table 2). 

Similarly, platinum accumulation increased in ABCC2-siRNA-transfected PANC-1 cells as compared with the PANC-1 cells transfected with the negative control siRNA. The cellular platinum accumulation after 2 h exposure to 25 µM and 100 µM of oxaliplatin in control-siRNA PANC-1 cells were 5.9 ± 0.3 and 32.6 ± 0.6 pmol/mg of protein respectively. Silencing MRP2 resulted in 6.5–15-fold increase in the platinum accumulation after 2 h exposure to 25 µM or 100 µM of oxaliplatin in ABCC2-siRNA PANC-1 cells (Table 2). 

### 2.5. ABCC2-siRNA Increased Oxaliplatin-Induced Apoptosis Rate

**Figure 4:**
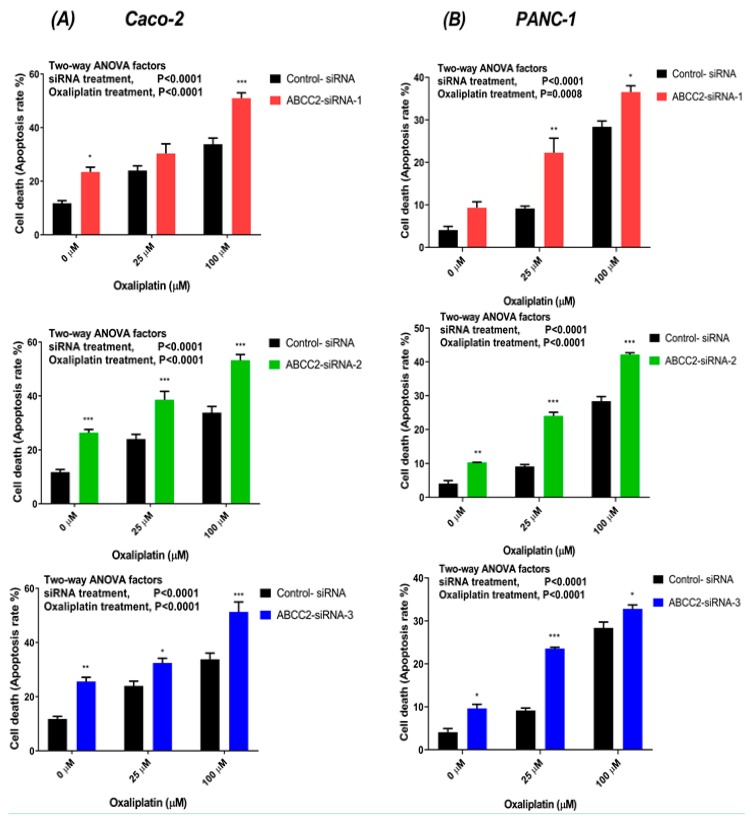
Oxaliplatin-induced apoptosis in (**A**) Caco-2 and (**B**) PANC-1 cells transfected with ABCC2-siRNAs and control-siRNA. The cells were treated with oxaliplatin (25 μM and 100 μM) for 2 h before stained with Annexin V-FITC/PI, and the rate of apoptosis was measured by flow cytometry. Data are presented as the mean of total apoptosis rate in percentage and standard errors of the mean of three independent experiments. Asterisks are P values (*, *p* < 0.05; **, *p* < 0.01; ***, *p* < 0.001) from Sidak post-tests that followed a two-way ANOVA.

The oxaliplatin-induced apoptosis was measured by flow cytometric detection of Annexin V-FITC binding to phosphatidylserine on the external cell surface of early apoptotic cells and PI-stained late apoptotic cells. These results showed that oxaliplatin induced an explicit increase of total apoptosis rate (the sum of early and late apoptosis) in a concentration-dependent manner. The control and ABCC2-siRNA-transfected Caco-2 and PANC-1 cells were treated with oxaliplatin (25 µM and 100 µM) for 2 h. 48-h after treatment, we observed increased apoptosis rate in the ABCC2-siRNA-transfected cells as compared to control-transfected cells. In Caco-2 cells treated with 25 µM oxaliplatin, the apoptosis rate increased by 5.8 ± 8% (ns), 16 ± 8.9% (*p* < 0.001) and 10.86 ± 4.6% (*p* = 0.01) in ABCC2-siRNA-1, ABCC2-siRNA-2, and ABCC2-siRNA-3, respectively, compared to negative control–transfected cells (Figure 4A). After 100 µM oxaliplatin treatment the apoptosis percentage increased by 16.2 ± 6.6% (*p* < 0.001), 20.4 ± 6.4% (*p* < 0.001) and 16.5 ± 8.4% (*p* < 0.001) for ABCC2-siRNA-1, ABCC2-siRNA-2, and ABCC2-siRNA-3, respectively (Figure 4A). 

After treatment with 25 µM oxaliplatin the apoptosis rate increased by 14 ± 1.4% (*p* < 0.001), 14.8 ± 1.7% (*p* < 0.001), and 14.2 ± 0.9% (*p* < 0.001) in ABCC2-siRNA-1-, ABCC2-siRNA-2-, and ABCC2-siRNA-3-transfected PANC-1 cells (Figure 4B). In PANC-1 cells treated with 100 µM oxaliplatin, the apoptosis rate increased by 7.1 ± 1.5% (*p* = 0.01), 9.2 ± 1.9% (*p* < 0.01), and 6.8 ± 1.3% (*p* < 0.01) in ABCC2-siRNA-1-, ABCC2-siRNA-2-, and ABCC2-siRNA-3-transfected cells, respectively (Figure 4B). Silencing MRP2 gene using ABCC2-siRNAs transfection enhanced the oxaliplatin-induced apoptosis rate in tested GI cancer cells.

### 2.6. Effect of Myricetin on Oxaliplatin Accumulation and Growth Inhibition

We recently reported that a model MRP2 inhibitor myricetin increased cellular platinum accumulation and oxaliplatin cytotoxicity in human HepG2 and PANC-1 cells [20]. A human colorectal cancer cell line, Caco-2, was used to assess the effect of myricetin on cellular oxaliplatin accumulation and sensitivity. Cells were treated with 50 μM oxaliplatin for 2 h in the presence and absence of 60 μM myricetin, followed by measurement of cellular platinum accumulation by ICP-MS. To measure the cell viability by MTT assay, cells were incubated with oxaliplatin at various concentrations for 2 h with or without 60 μM myricetin. Myricetin slightly but significantly increased platinum accumulation and reduced oxaliplatin-induced growth inhibition IC_50_ values in Caco-2 cells (Table 3).

### 2.7. Oxaliplatin Stimulated the ATPase Activity of MRP2

**Figure 5:**
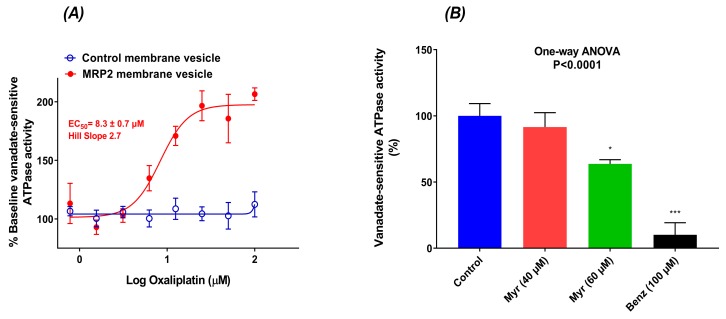
(**A**) Effects of oxaliplatin (0.78 µM to 100 µM) on vanadate-sensitive ATPase activity in plasma membrane vesicles from sf9 cells overexpressing human MRP2 (MRP2 MVs, ●) and control MVs (○). The data were normalised to the baseline vanadate-sensitive activity of the reaction. All data were expressed as mean ± standard errors of the mean, *n* = 4. (**B**) Effect of myricetin (40 µM and 60 µM) and benzbromarone (100 µM) on oxaliplatin (25 µM) induced vanadate-sensitive ATPase activity. All data are represented as mean ± standard errors of the mean, *n* = 4. Asterisks are *p* values (*, *p* < 0.05; ***, *p* < 0.001) from Dunnett’s post hoc test that followed one-way ANOVA for comparisons of all samples to basal vanadate-sensitive ATPase activity sample. Myr and Benz represent myricetin and benzbromarone, respectively.

The interaction between oxaliplatin and MRP2 ATPase was investigated using the membrane vesicles prepared from Sf9 cells overexpressing human MRP2 or control Sf9 cells. As shown in Figure 5A, oxaliplatin stimulated the vanadate-sensitive MRP2 ATPase activities in a concentration-dependent manner, with maximal stimulation of two-fold of the basal activity, an EC_50_ value of 8.3 ± 0.7 µM, and a Hill slope of 2.7 in MRP2 membrane vesicles. Oxaliplatin had no apparent stimulation effects on vanadate-sensitive ATPase activities in control membrane vesicles.

To study whether MRP2 inhibitors inhibit the oxaliplatin stimulated vanadate-sensitive MRP2 ATPase activity, we used myricetin and benzbromarone in ATPase reaction. Benzbromarone, a known MRP2 inhibitor, is used as a positive control. In the presence of 25 μM of oxaliplatin, the oxaliplatin stimulated MRP2 ATPase activity was significantly reduced by 40% and 90% after incubation with 60 µM myricetin and 100 µM benzbromarone respectively (Figure 5B). Myricetin at 40 μM had no apparent effect on oxaliplatin induced MRP2 ATPase activity.

## 3. Discussion

Based on robust evidence from multiple randomised controlled trials, oxaliplatin and its combination regimens is clinically important for the treatment of colorectal, pancreatic, and other gastrointestinal cancers [1,21,22,23,24,25,26,27]. Although some patients benefit from oxaliplatin-based treatment, some patients fail to respond to treatment. There is still no clear method to distinguish gastrointestinal tumours that will or will not respond to oxaliplatin-based regimens. It is therefore important to identify biomarkers that predict resistance to oxaliplatin and alternative treatment approaches for patients with poor outcomes from oxaliplatin-based treatment. We have recently identified MRP2 as a targetable factor limiting oxaliplatin accumulation and response in gastrointestinal cancer [20].

In the current study, silencing MRP2 by siRNA was used to further clarify the contribution of MRP2 to oxaliplatin resistance in Caco-2 and PANC-1 cells with endogenous MRP2 overexpression (Figure 1). Our in vitro studies provided insights into mechanisms underlying transport of oxaliplatin by MRP2, resulting in extrusion of oxaliplatin from cancer cells and thus leading to decreased cellular platinum accumulation and cytotoxicity of oxaliplatin on cancer cells. Previously, we demonstrated that inhibition of MRP2 by myricetin or siRNA knockdown increased the accumulation of platinum and sensitivity to oxaliplatin in HepG2 cells. In our analysis of a clinical gene expression dataset, ABCC2 was the only one of 18 oxaliplatin transporter candidate genes with differential tumour expression between CRC patients who did or did not respond to oxaliplatin-based chemotherapy [20]. In our current study, we extended these findings to show effects of silencing MRP2 by siRNA in other human gastrointestinal cancer cell lines (Caco-2 and PANC-1) with endogenous overexpression of MRP2. This study provided direct evidence that silencing MRP2 by siRNA reduced the mRNA and surface protein expression of MRP2 (Figure 2, Figures S1 and S2). Inhibition of MRP2 by siRNA knockdown increased the accumulation (Table 2) and cytotoxicity of oxaliplatin in Caco-2 and PANC-1 cells (Table 1 and Figure 3). In our present work, silencing MRP2 by siRNA also resulted in an increase in oxaliplatin-induced total apoptosis rate in Caco-2 and PANC-1 cells (Figure 4, Figures S3 and S4). These results suggested that increased platinum accumulation elevated the total apoptosis rate and increased sensitivity to oxaliplatin. Interestingly, knockdown of MRP2 by siRNA alone also increased the apoptosis rate in Caco-2 cells (Figure 4A), which is consistent with a previous report in resistant HepG2 cells [16]. However, silencing MRP2 along had no apparent effects on total apoptosis rate in PANC-1 cells (Figure 4A). Although ABC transporters mediated cytotoxic drug efflux affects the resistance of cancer cells to apoptosis in the context of therapy, several reports provide evidence that ABC transporters might also protect cells from apoptosis independently of cytotoxic drug efflux [28,29,30,31,32]. Further studies are required to better understand the molecular mechanisms of the potential roles of MRP2 in the protection of apoptosis in Caco-2 cells. 

Our present results (Table 3) suggest human colorectal cancer Caco-2 cells accumulated much less platinum (15–23%) compared with other human gastrointestinal cancer lines (HCT116, MiaPACA-2, WiDr, SW620, and HT29) [20] during in vitro exposure to oxaliplatin (50 μM) for two hours. A model MRP2 inhibitor myricetin significantly increased platinum accumulation and oxaliplatin sensitivity in HepG2 and PANC-1 cells [20]. While Caco-2 cells accumulated similarly low platinum as HepG2 and PANC-1 cells, myricetin only slightly (but significantly) enhanced platinum accumulation and oxaliplatin cytotoxicity in Caco-2 cells. This suggests myricetin might affect other rate-limiting factors for platinum accumulation specifically in Caco-2 cells. Myricetin is a potent inhibitor for a wide range of protein kinases including tyrosine kinase [33]. A recent study reported that inhibition of tyrosine kinase in vivo diminished organic cation transporter 2 (OCT2) activity through blocking OCT2 tyrosine phosphorylation, significantly mitigating oxaliplatin-induced acute sensory neuropathy [34]. OCT2, an important oxaliplatin transporter [10], also endogenously expressed on Caco-2 cells [35]. However, whether myricetin can inhibit OCT2 tyrosine phosphorylation and thus its activity remains unknown in Caco-2 cells. 

This study also provides experimental proof of the interaction of MRP2 ATPase activity with oxaliplatin. Like other ABC transporters, MRP2 uses ATP as a source of energy to transport substrate across cell membranes. In the ATPase assay, stimulation of ATPase activity by a test compound is coupled to the actual transport process and is therefore indicative of the substrate-type relationship [36]. In our MRP2 ATPase study, oxaliplatin stimulated the basal vanadate-sensitive ATPase activity in the Sf9/MRP2 membrane vesicles, and the stimulatory effect of oxaliplatin was best fit with a sigmoidal dose-response model with an EC50 value of 8.3 ± 0.7 µM. Benzbromarone, a well-defined MRP2 inhibitor [37,38], almost completely abolished oxaliplatin stimulated MRP2 ATPase activity. However, oxaliplatin showed no effect on ATPase activity in the control membrane vesicles, ruling out the possibility that oxaliplatin might stimulate wild-type ABC transporters expressed in Sf9 cells. Together, these observations suggest that oxaliplatin is a substrate of MRP2, which is consistent with our previous results obtained from direct transport assay using MRP2 membrane vesicles [15]. MRP2 is localized at the canalicular membrane of the liver and apical side of kidney proximal tubule cells and can play a significant role in the disposition and excretion of its substrate drugs, their efficacy and toxicity [39]. Approximately half of the dose of oxaliplatin is excreted in the urine, and renal platinum clearance was similar to or exceeded the average human glomerular filtration [40]. Further studies are warranted to verify the contribution of MRP2 on oxaliplatin pharmacokinetics. 

The Hill coefficient was 2.7 in the sigmoidal curve of oxaliplatin stimulated ATPase activity (Figure 5A), suggesting the existence of at least two substrate binding sites in the MRP2 protein, which is consistent with the previous publications [38,41,42]. Since the stimulation effect of oxaliplatin was a sigmoidal-shaped curve, we postulate that the ATPase activity increases with increasing concentration until it reaches a plateau because the relative affinity of oxaliplatin for the second inhibitory site is lower than the transport site by at least several orders of magnitude. Hence, oxaliplatin may have a stronger affinity for interacting with MRP2 at the transport site, which stimulates the MRP2 ATPase activity. The presence of a second binding site may also imply potential allosteric interactions of MRP2-mediated oxaliplatin transport. Allosteric stimulation of MRP2 activities is common, and the stimulators could be even a model MRP2 inhibitor (for another MRP2 substrate with a different binding site), poor MRP2 substrates, or non-substrates [41,42]. Our results also demonstrated that a model MRP2 inhibitor myricetin [43,44] inhibited the oxaliplatin-induced MRP2 ATPase activity in a concentration-dependent manner, but it appears to be a weak inhibitor. These findings now encourage further in vivo translation of the oxaliplatin-based treatment with alternative methods of genetic manipulation and more selective and potent pharmacological inhibitors capable of complete disruption of MRP2 function.

Currently, the numbers of clinical tumour gene expression datasets with oxaliplatin tumour resistance data are very limited. In our analysis of a clinical gene expression dataset, we showed that ABCC2 was the only one of 18 oxaliplatin transporter candidate genes with differential tumour expression between CRC patients who did or did not respond to oxaliplatin-based chemotherapy [20]. One recent large-scale, multicentre clinical trial showed ABCC2 genetic polymorphisms are associated with worse overall survival in stage I pancreatic cancer, but there is no detailed information regarding the therapies which the patients had received [45]. Further interrogation of a broader range of clinical datasets is now required to bolster clinical correlations with ABC/SLC transporter gene sequence and mRNA expression level variation. 

## 4. Materials and Methods 

### 4.1. Materials

The cell cultures media DMEM, RPMI 1640 and Opti-MEM, trypLE express, penicillin-streptomycin, L-glutamine, and transfection reagents including stealth RNAi, siRNA, and Lipofectamine RNAiMAX were from Life Technologies (Auckland, New Zealand). The apoptosis kit, myricetin, and CDCFDA (5,6-Carboxy-2’7’-Dichlorofluorescein diacetate) were from Invitrogen (Carlsbad, CA, USA). Foetal bovine serum (FBS) was from MediRay (Auckland, New Zealand). Anti-MRP2 mouse monoclonal antibody (M2 III-6) and goat anti-mouse IgG H&L (Alexa Fluor 488) secondary antibody were procured from Abcam (Cambridge, UK). The PCR kit was from Roche Diagnostics (Basel, Switzerland). Thallium and platinum standards, and MRP2 ATPase assay kit was from Sigma-Aldrich (Auckland, New Zealand). Oxaliplatin (Actavis, Auckland, New Zealand) stock solution at 5mg/ml was prepared by dissolving 100 mg powder into 20 mL milliQ water.

### 4.2. Cell Culture

The human gastrointestinal cancer cell lines, Caco-2 and PANC-1, were from American Type Culture Collection (ATCC, Manassas, VA, USA). Caco-2 and PANC-1 cell lines were cultured and maintained in the RPMI 1640 medium. The cultured media were supplemented with 10% (v/v) FBS, 2 mmol/L L-glutamine, 100 units/mL of penicillin, and 100 µg/mL streptomycin, in a humidified atmosphere of 5% carbon dioxide at 37 °C.

### 4.3. ATPase Assay

Membrane ATPase activity was measured by utilising membranes from Sf9 cells overexpressing MRP2 or control membrane mock transfected. For the activation studies, both control and MRP2 membrane vesicles (8 µg/well) were incubated with 2 mM glutathione, 10 mM MgATP and oxaliplatin at various concentrations (0.78 μM to 100 μM) in the presence and absence of 1.5 mM sodium orthovanadate at 37 °C for 20 min. For inhibition study, an MRP2 inhibitor myricetin (40 µM and 60 µM) and a well-defined MRP2 ATPase inhibitor benzbromarone (100 µM) were used to block oxaliplatin-activated MRP2 ATPase activity. The reaction was stopped by adding a developer solution and the colour was developed by adding a blocker solution at room temperature. The absorbance at 620 nm was measured on a SPARK Tecan 10M microplate reader (Mannedorf, Switzerland). ATPase activities were determined as the difference of inorganic phosphate liberation measured in the presence and absence of 1.5 mM sodium orthovanadate. The EC_50_ is defined, as the concentration needed to reach 50% of the maximal stimulation. For each experiment, all the concentrations of oxaliplatin were tested in quadruplicate. 

### 4.4. siRNA Transfection

Double strand siRNAs (siRNA-1, siRNA-2 and siRNA-3) targeting ABCC2 gene [20] were transfected into Caco-2 and PANC-1 cells using Lipofectamine RNAiMAX. A non-targeting negative stealth siRNA (scrambled) was used as a negative control. Reverse and forward transfection were used for transfecting Caco-2 cells and PANC-1 cells, respectively. For reverse transfection, cells were seeded into a 12-well plate at a density of 1.5 × 10^5^ cells per well and transfected with different ABCC2 siRNA subtypes at 40 pmol using Lipofectamine RNAiMAX. For forward transfection, one day before transfection, 10^5^ cells were plated in the 12-well plate, and next day, cells were transfected with ABCC2 siRNA subtypes at 30 pmol using Lipofectamine RNAiMAX. After 48 h, experiments were performed to assess effects on MRP2 expression and function, platinum accumulation and oxaliplatin-induced growth inhibition. The sequences clone ID NM_000392.4 of validated stealth ABCC2 siRNAs are listed below.

a. ABCC2-siRNA-1: 5’-GAU CAU GAA UGA GAU UCU UAG UGG A-3’
  5’-UCC ACU AAG AAU CUC AUU CAU GAU C-3’
b. ABCC2-siRNA-2: 5’-CCA GCA AAG GCA AGA UCC AGU UUA A-3’
  5’-UUA AAC UGG AUC UUG CCU UUG CUG G-3’
c. ABCC2-siRNA-3: 5’-ACC AAG ACA UUA GUG AGC AAG UUU G-3’
  5’-CAA ACU UGC UCA CUA AUG UCU UGG U-3’

### 4.5. Expression of Transporter Genes

Total RNA was isolated from cultured cells using an RNeasy Mini kit (Qiagen, Valencia, CA, USA) according to the manufacturer’s instructions. The total RNA was quantified using Qubit® 2.0 Fluorometer (Invitrogen). Primer sequences as listed in Table 4 were purchased from IDT (Integrated DNA Technology, Coralville, IA, USA). Quantitative real-time PCR was performed with a LightCycler 480 Instrument II (Roche Diagnostics, Auckland, New Zealand) using LightCycler-FastStart DNA Master SYBR Green 1 Master Mix and gene-specific primers at 180 nM. The reaction conditions were as follows: 95 °C for 10 min, followed by 45 cycles at 95 °C for 15 s, at 58 °C for 30 s, and 72 °C for 30 s. ABCC2 mRNA expression in each sample was normalised to the reference gene GAPDH (Figure S5 shows the stability data of GAPDH expression after siRNA transfection), and results were analysed using the comparative threshold cycle method. 

### 4.6. MRP2 Surface Staining

The cell surface expression of MRP2 on Caco-2 and PANC-1 cells was semi-quantified by flow cytometry. After transfecting cells with ABCC2-siRNA subtypes and control-siRNA, the cells were washed with warm PBS, trypsinised, resuspended in PBS, and aliquoted at 2 × 10^5^ cells into Eppendorf tubes. After fixing in 1% paraformaldehyde (PFA), the cells were washed, permeabilised with 0.2% saponin, blocked with 5% BSA/PBS, and finally stained with primary (MRP2 M2 III-6 or an IgG2a isotype control) and secondary antibody (goat anti-mouse IgG H&L (Alexa Fluor 488)) at room temperature. The cells were washed thrice with PBS between each steAfter secondary antibody incubation, cells were then washed and resuspended in 200 µL of 1% PFA/PBS. Fluorescence was detected at 488 nm excitation and 530/30 nm emission wavelength. Data is presented as a % of the MFI of control = [(MFI of ABCC2 siRNA − MFI of isotype control)/(MFI of control siRNA − MFI of isotype control)] × 100.

### 4.7. MRP2 Substrate Accumulation 

Steady-state cellular accumulation of an MRP2 substrate, 5,(6)-carboxy-2’7’-dichlorofluorescein (CDCF) were determined using a flow cytometer (Beckman Coulter, MoFlo XDP). Transfected and control cells of density 500,000 cells/mL were incubated with 5 μM CDCFDA for 10 min at 37 °C. After the incubation, the cells were washed with ice-cold PBS twice and resuspended in PBS and kept at 4 °C until analysis. The samples were analysed with a standard laser for excitation at 488 nm and a bandpass filter at 530 nm to detect fluorescence. Cells were gated to exclude dead cells, cellular debris and cell doublets. The mean fluorescence intensity was determined using Kaluza Flow cytometry software analysis (Beckman Coulter, Brea, CA, USA).

### 4.8. Platinum Accumulation 

The cellular accumulation of platinum in the transfected cells was determined by Inductively Coupled Plasma mass spectrometry (ICP-MS) by measuring the platinum concentration in cell lysates using thallium as an internal standard. After transfection of Caco-2 and PANC-1 cells, cells were seeded at 250,000 cells per well in six-well plates and grown in the normal growth medium until around 80% confluent. The cells were then incubated with 25 µM of oxaliplatin for 2 h. At the end of treatment duration, cells were washed three times with ice-cold PBS, then dried in room air for 30 min and dissolved in 300 μL of 70% nitric acid for 2 h to digest the cells. A 300 μL aliquot of the digested cells was added to a 96-well plate to determine protein content using absorbance-based BSA protein quantification. After the protein assay, the aliquots were transferred to 5 mL screw-top vials for further digestion in 70% nitric acid at room temperature for overnight before heating at 95 °C for 2 h. The digested cells were diluted in 50 ppb of thallium before ICP-MS analysis using Varian 820MS ICP-MS (Agilent Technologies Inc., Santa Clara, CA, USA). The signal/noise optimisation was carried out prior to each run using a tuning solution of platinum and thallium. Then, the platinum concentration of each sample was calculated from platinum to thallium count ratio using the standard curve method and a calibration curve made from standard solutions at the desired platinum concentration made up in the same matrix as the samples. Quality control samples at three different concentrations were included for determining the accuracy, precision, and reliability of each run. An ICP-MS run was acknowledged only if the standard was linear and quality control samples over the standard curve were within 85% to 115% accuracy values. The limit of detection and lower limit of quantification were 0.3 ppb and 1 ppb, respectively. 

### 4.9. Cytotoxicity Assay

Cells were seeded at a density of 5000 cells per well in 96-well plates and grown overnight at 37 °C and 5% CO_2._ After overnight incubation, cells were incubated with culture medium containing a series of concentrations of oxaliplatin for 2 h at 37 °C with 5% CO_2_. To terminate the drug exposure, the drug-containing medium was replaced with fresh drug-free medium and cells were then incubated for 72 h under usual conditions before the MTT assay was performed. The cell viability at different drug concentration was quantified by measuring photometric absorbance at 570 nm normalised to the mean absorbance of control untreated cells. The IC_50_ values for oxaliplatin-induced growth inhibition were determined using nonlinear regression in GraphPad Prism 7.0 software (GraphPad Software, Inc., La Jolla, CA, USA).

### 4.10. Annexin-V-FITC Apoptosis Assay

Annexin V-FITC apoptosis detection kit was used to assess the apoptotic effect of oxaliplatin in MRP2 silencing Caco-2 and PANC-1 cells. The transfected cells were double stained with annexin V‑fluorescein isothiocyanate (FITC) and propidium iodide (PI) in the dark for 15 min at room temperature according to the manufacturer’s instructions. Cells in early apoptosis stage were annexin V^+^/PI^−^, whereas cells in the late apoptotic stage were annexin V^+^/PI^+^. Cells which were annexin V^−^/PI^−^ were considered as viable cells. Dead cells, cellular debris and doublets were excluded using forward and side scatter. Flow cytometric data were collected on a MoFlo XDP instrument and analysed with Kaluza software (Beckman Coulter, Brea, CA, USA).

### 4.11. Statistical Analysis

The values were expressed as a mean ± standard errors of the mean. All the results were from at least three independent experiments. Statistical analyses were performed using the GraphPad Prism 7. Multiple comparisons between control and different treatment groups were analysed using analysis of variance (ANOVA) with a post-hoc test. *p*-value < 0.05 was considered as significant, whereas *p*-values < 0.01 and < 0.001 were considered as highly significant.

## 5. Conclusions

Taken together, our in vitro study shows the interaction of oxaliplatin with MRP2, and silencing MRP2 in Caco-2 and PANC-1 cells increased platinum accumulation and oxaliplatin sensitivity. We have previously shown a similar pattern of results in HepG2 cells [20]. Thus, high endogenous expression levels of the MRP2 transporter may compromise oxaliplatin efficacy by decreasing platinum accumulation in some gastrointestinal cancer cells. We propose that MRP2 could be a potential marker of chemotherapy response to stratify patients for either oxaliplatin alone or in combination with MRP2 modulation treatment. 

## Figures and Tables

**Table 1:** Oxaliplatin-induced growth inhibition: Comparison between control-siRNA and ABCC2-siRNAs-transfected cells.

Cell Lines	Transfected Cells	IC50 * (µM) (Mean ± SEM)	*p*-Value
Caco-2	Control-siRNA	13.82 ± 1.21	-
	ABCC2-siRNA-1	7.75 ± 0.08	0.0001
	ABCC2-siRNA-2	8.4 ± 0.13	0.0001
	ABCC2-siRNA-3	7.03 ± 0.76	0.0001
PANC-1	Control-siRNA	35.13 ± 3.19	-
	ABCC2-siRNA-1	16.48 ± 0.53	0.018
	ABCC2-siRNA-2	11.91 ± 0.25	0.027
	ABCC2-siRNA-3	14.60 ± 0.72	0.012

* Data shown are mean ± standard errors of the mean from three independent experiments.

**Table 2:** Cellular accumulation of multidrug resistance protein 2 (MRP2) substrate, 5(6)-carboxy-2,’7’-dichlorofluorescein (CDCF) and oxaliplatin-derived platinum in control and ABCC2-siRNA-transfected cells ^#^.

Cell Lines	siRNA Transfection	CDCF Accumulation (% of Control)	Platinum Accumulation (pmol/mgprotein)	
			25 μM Oxaliplatin	100 μM Oxaliplatin
Caco-2	Control-siRNA	100 ± 8.4	5.8 ± 0.3	105.2 ± 0.4
	ABCC2-siRNA-1	133.1 ± 7.1 **	10.6 ± 2.6	128.8 ± 3.0
	ABCC2-siRNA-2	152.6 ± 3.7 **	11.4 ± 1.0 *	152.5 ± 0.7 *
	ABCC2-siRNA-3	145.5 ± 4.5 **	11.2 ± 1.5 *	180.7 ± 24.5 **
PANC-1	Control-siRNA	100 ± 3.89	5.92 ± 0.26	32.6 ± 0.6
	ABCC2-siRNA-1	153.6 ± 6.3 **	78.0 ± 2.4 **	260.6 ± 2.7 **
	ABCC2-siRNA-2	154.1 ± 10.0 **	84.3 ± 10.0 **	212.5 ± 14.8 **
	ABCC2-siRNA-3	148.8 ± 3.9 **	72.3 ± 11.3 **	217.4 ± 50.5 **

^#^ Data are presented as the mean ± standard errors of the mean. Asterisks are *p* values (*, *p* < 0.05; **, *p* < 0.01) from Dunnett’s post hoc test that followed one-way ANOVA for comparisons of all ABCC2-siRNA transfected samples to the negative control.

**Table 3:** Effect of myricetin on oxaliplatin accumulation and cytotoxicity in Caco-2 cells *.

Cell Lines	Cellular Platinum Accumulation (pmol/mg Protein)		*p*-Value	IC50 (µM)		*p*-Value
	Myricetin	Control		Myricetin	Control	
Caco-2	87 ± 3.35	74 ± 2.07	0.03	7.72 ± 0.36	10.4 ± 0.79	0.03

* Data shown are mean ± standard errors of the mean from three independent experiments.

**Table 4:** Primers used for RT-qPCR.

Target Genes	Forward Primers (5’-3’)	Reverse Primers (5’-3’)	Reference *
ABCC2	AATCAGAGTCAAAGCCAAGATGCC	TAGCTTCAGTAGGAATGATTTCAGGAGCAC	[46]
GAPDH	GCACCGTCAAGGCTGAGAAC	GCCTTCTCCATGGTGGTGAA	[47]

* The primers used in the current study are from the published papers.

## Supplementary Materials

The following are available online at https://www.mdpi.com/2072-6694/11/9/1330/s1, Figure S1: MRP2 surface immunostaining flow cytometry histogram data in Caco-2 cells, Figure S2: MRP2 surface immunostaining flow cytometry histogram data in PANC-1 cells, Figure S3: Oxaliplatin-induced apoptosis rate in MRP2 silenced Caco-2 cells, Figure S4: Oxaliplatin-induced apoptosis rate in MRP2 silenced PANC-1 cells, Figure S5: Effects of control and ABCC2-siRNA transfection on the Cp values of the reference gene GAPDH in Caco-2 (A) and PANC-1 (B) cells.
