# Supplementary material for: Transport-Mediated Oxaliplatin Resistance Associated with Endogenous Overexpression of MRP2 in Caco-2 and PANC-1 Cells
Source: Cancers (Basel). 2019 Sep 8;11(9):1330. doi: 10.3390/cancers11091330 (PMC6770320; doi:10.3390/cancers11091330)
Supplement: Supplementary file 1 [file cancers-11-01330-s001.pdf]

Supplementary Materials: Transport-Mediated Oxaliplatin Resistance Associated with Endogenous Overexpression of MRP2 in Caco-2 and PANC-1 Cells

Riya Biswas, Piyush Bugde, Ji He, Fabrice Merien, Jun Lu, Dong-Xu Liu, Khine Myint, Johnson Liu, Mark Mckeage and Yan Li

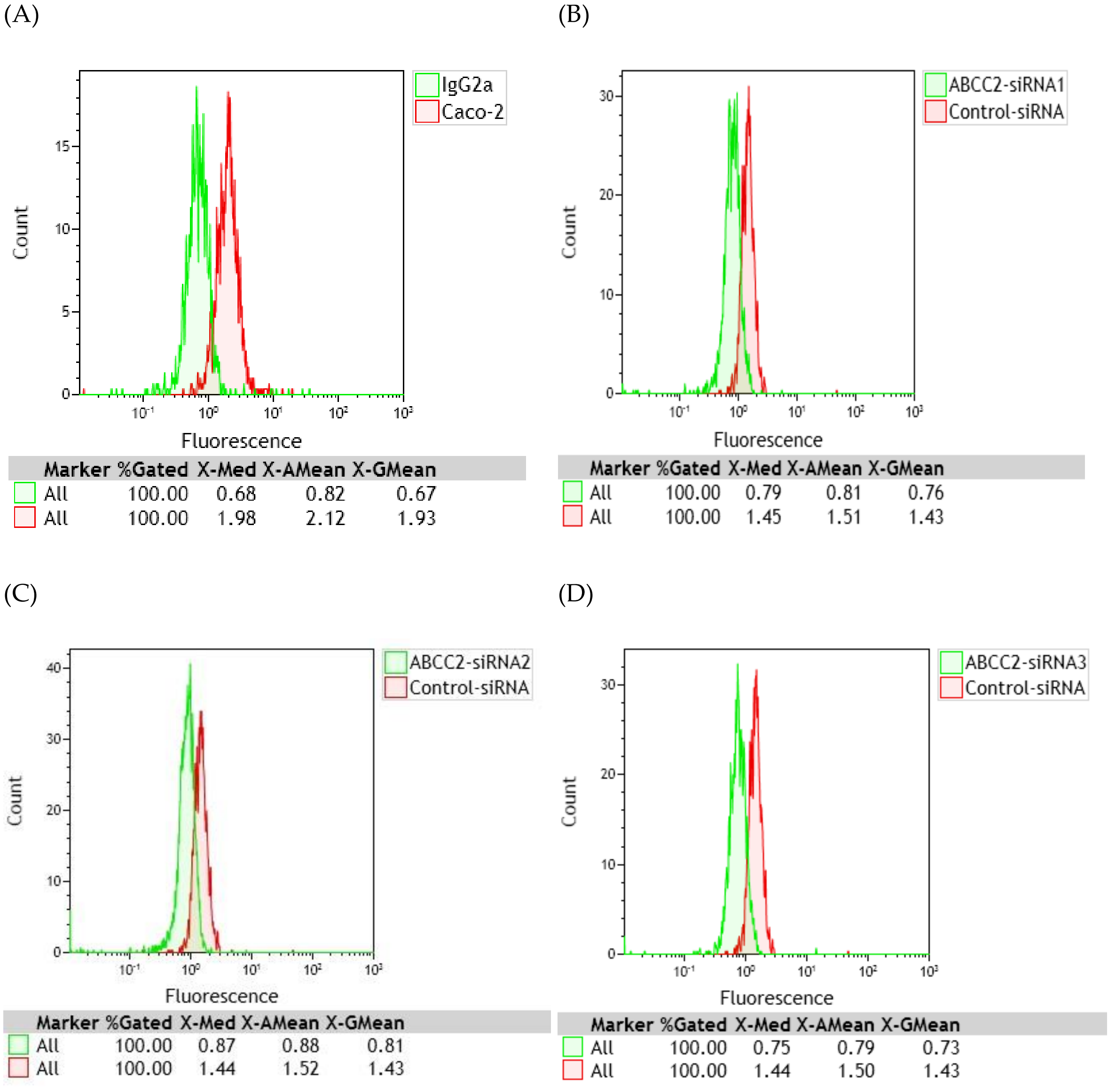

(A)

Marker	%Gated	X-Med	X-AMean	X-GMean
All	100.00	0.68	0.82	0.67
All	100.00	1.98	2.12	1.93

(B)

Marker	%Gated	X-Med	X-AMean	X-GMean
All	100.00	0.79	0.81	0.76
All	100.00	1.45	1.51	1.43

(C)

Marker	%Gated	X-Med	X-AMean	X-GMean
All	100.00	0.87	0.88	0.81
All	100.00	1.44	1.52	1.43

(D)

Marker	%Gated	X-Med	X-AMean	X-GMean
All	100.00	0.75	0.79	0.73
All	100.00	1.44	1.50	1.43

Figure S1. MRP2 surface staining flow cytometry histogram data in Caco-2 cells. Data represent flow cytometry histogram of cell surface staining using the ant-MRP2 primary antibody and isotype control IgG2a on Caco-2 and siRNA transfected Caco-2 cells. Graphs show the fluorescence intensity in **(**A) Caco-2 cells stained with MRP2 antibody and IgG2a stained cells; and (B, C, D) ABCC2-siRNAs transfected Caco-2 cell compared with control-siRNA cells. Both the primary antibody and isotype control were labelled with Alexa Fluor 488 secondary antibody. The x-axis is the fluorescence signal intensity in the FL1 blue laser channel displayed in a liner log scale. The y-axis represents the cell counts.

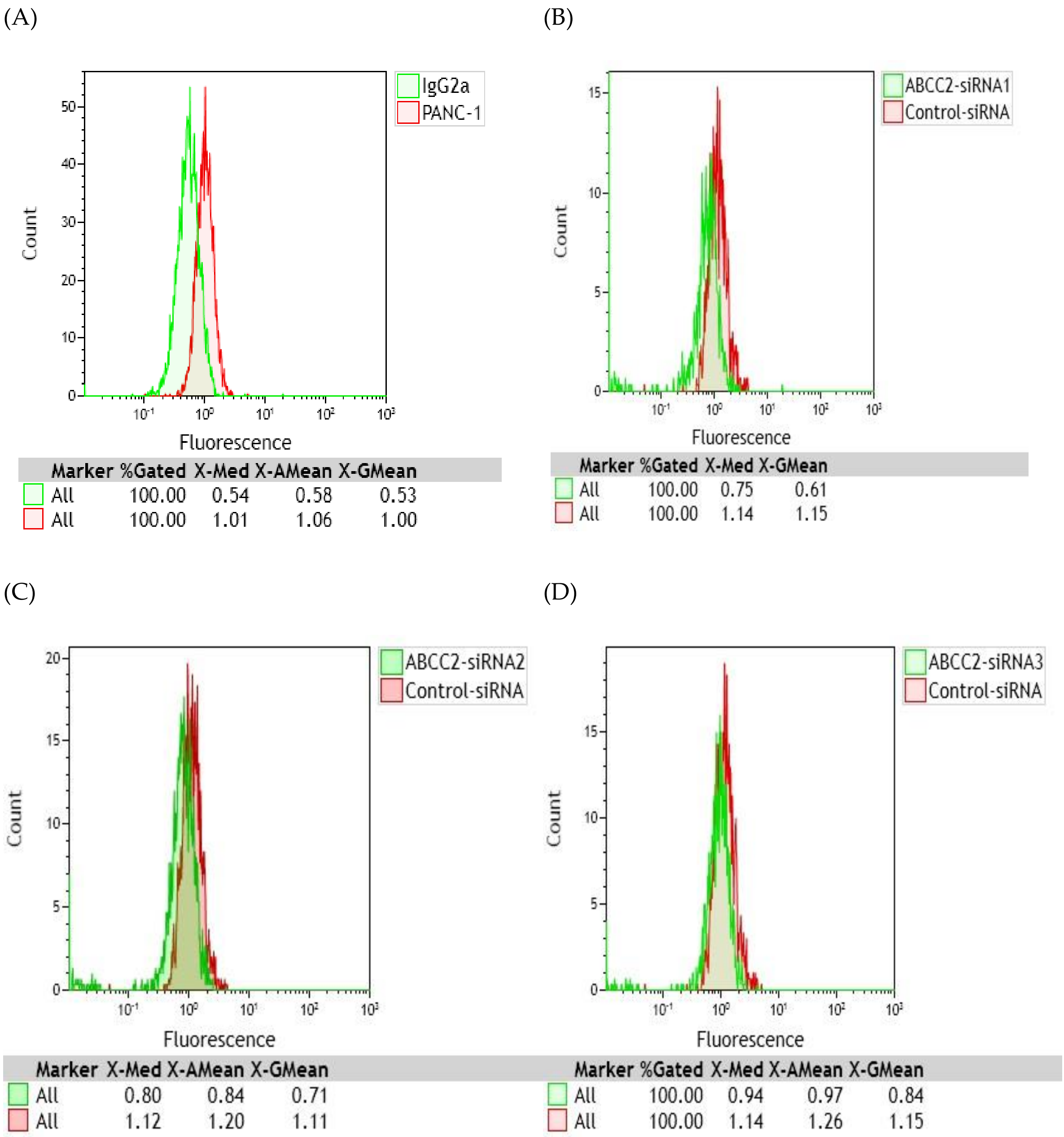

Figure S2. MRP2 surface staining flow cytometry histogram data in PANC-1 cells. Data represent flow cytometry histogram of cell surface staining using the ant-MRP2 primary antibody and isotype control IgG2a on PANC-1 and siRNA transfected PANC-1 cells. Graphs show the fluorescence intensity in **(**A) PANC-1 cells stained with MRP2 antibody and IgG2a stained cells; and (B, C, D) ABCC2-siRNAs transfected PANC-1 cell compared with control-siRNA cells. Both the primary antibody and isotype control were labelled with Alexa Fluor 488 secondary antibody. The x-axis is the fluorescence signal intensity in the FL1 blue laser channel displayed in a liner log scale. The y-axis represents the cell counts.

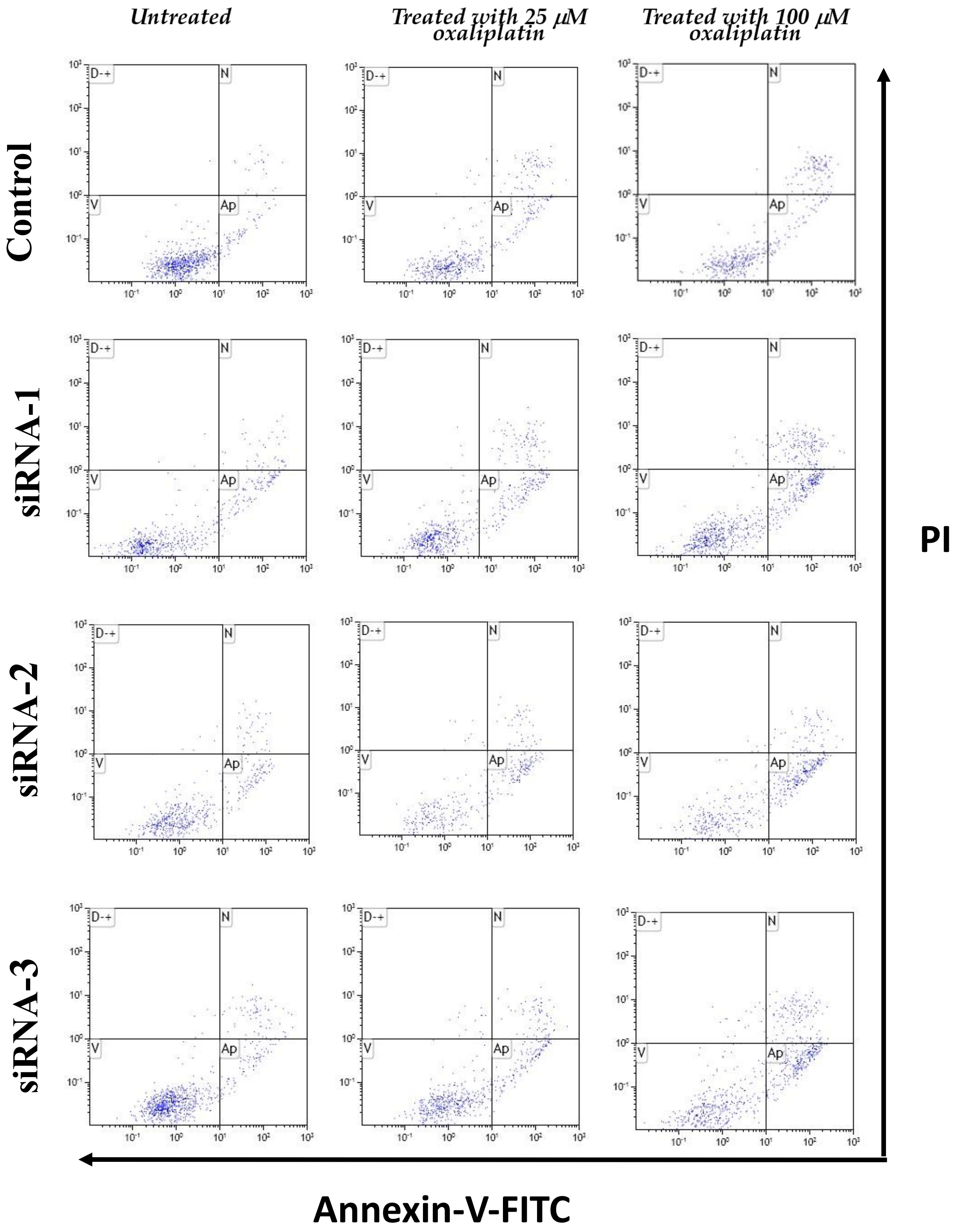

Figure S3. Oxaliplatin-induced apoptosis rate in MRP2 silenced Caco-2 cells. Caco-2 cells transfected with control and ABCC2-siRNAs were treated with oxaliplatin at different concentrations (0, 25 and 100 μM) for 2 hrs. Cells were then incubated in blank complete medium for 48 hrs and subsequently stained with Annexin-V-FITC and PI. The fluorescence intensity was measured by flow cytometry. Viable cells (V) are both Annexin-V and PI negative. At an early stage of apoptosis (Ap), the cells bind with only Annexin-V. At the late stage of apoptosis (N), the cells bind with both Annexin-V FITC and PI.

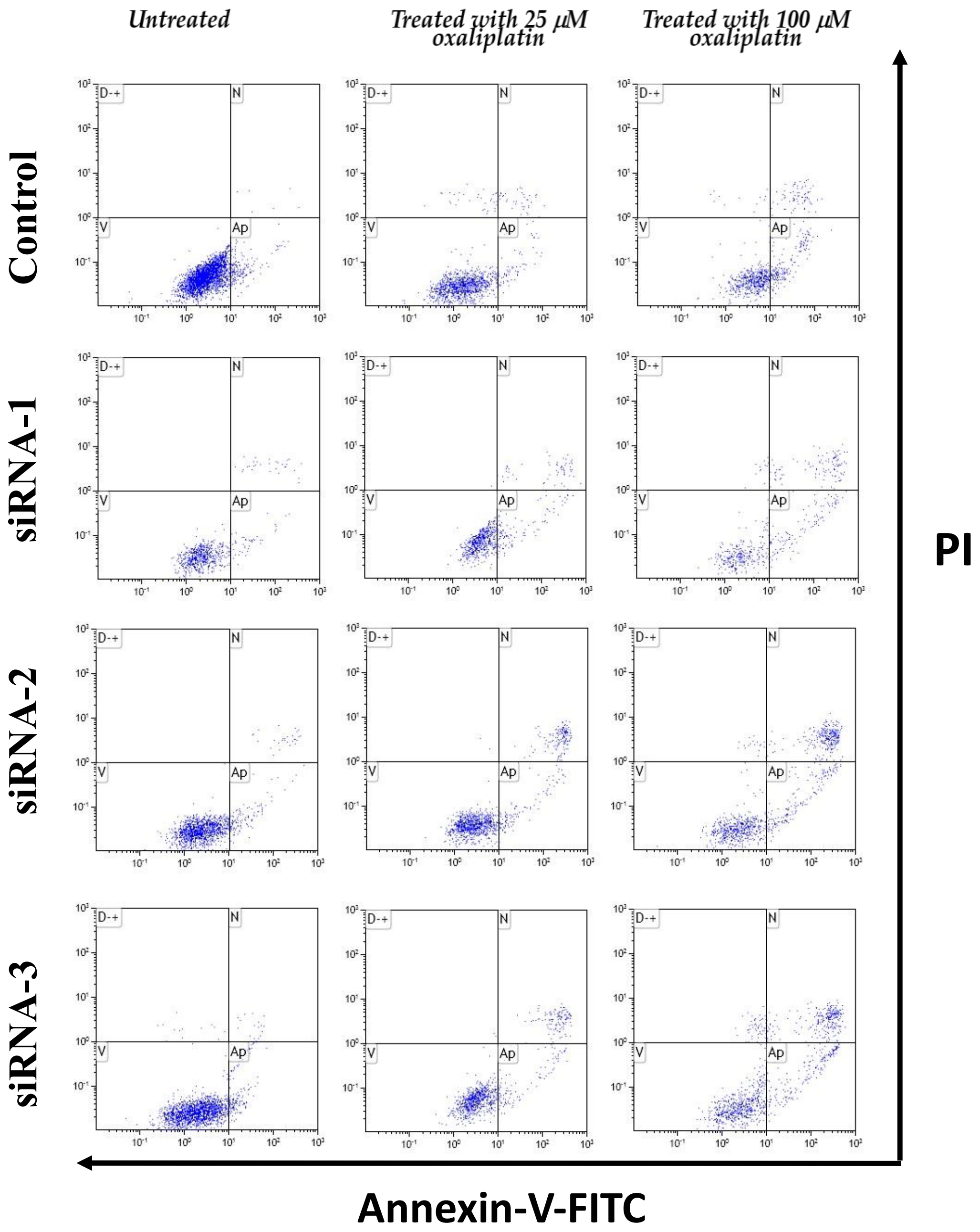

Figure S4. Oxaliplatin-induced apoptosis rate in MRP2 silenced PANC-1 cells. PANC-1 cells transfected with control and ABCC2-siRNAs were treated with oxaliplatin at different concentrations (25 and 100 µM) for 2 hrs. Cells were then incubated in blank complete medium for 48 hrs and subsequently stained with Annexin-V-FITC and PI and their fluorescence was measured by flow cytometry.

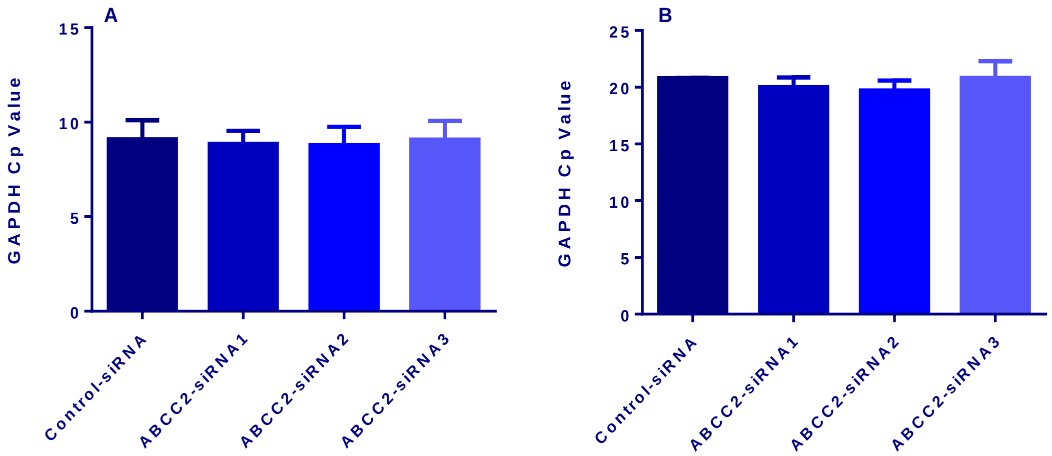

Figure S5. Effects of control and ABCC2-siRNA transfection on the Cp values of the reference gene GAPDH in Caco-2 (A) and PANC-1 (B) cells. All data were expressed as mean ± SEM from three independent experiments. No significant differences were detected from Dunnett's post hoc test that followed one-way ANOVA for comparisons of all ABCC2-siRNA samples to the negative control.

© 2019 by the authors. Submitted for possible open access publication under the terms and conditions of the Creative Commons Attribution (CC BY) license (http://creativecommons.org/licenses/by/4.0/).
